# REhabilitation of Dysphagia with ACupuncture and auricular Therapy after severe acquired brain injury (REDACT study): a double-blind randomized controlled trial protocol

**DOI:** 10.3389/fneur.2026.1724043

**Published:** 2026-02-24

**Authors:** Martina Calamini, Agnese De Nisco, Francesca Draghi, Francesca Cecchi, Paola Graziani, Antonello Grippo, Claudio Macchi, Daniela Maccanti, Annamaria Romoli, Silvia Pancani, Carmelo Guido, Bahia Hakiki

**Affiliations:** 1 School of Specialization in Physical and Rehabilitation Medicine, University of Florence, Florence, Italy; 2 IRCCS Fondazione Don Carlo Gnocchi, Florence, Italy; 3 Department of Experimental and Clinical Medicine, University of Florence, Florence, Italy; 4 Head and Neck and Robotic Surgery, Azienda Ospedaliero Universitaria Careggi, Florence, Italy; 5 SODc Neurophysiopathology, Department Neuromuscolo-Scheletrico e degli Organi di Senso, Azienda Ospedaliero Universitaria Careggi, Florence, Italy; 6 Regional Reference Center for Traditional Chinese Medicine, Corporate Coordination Center for Complementary Medicine, AUSL Toscana Centro, Florence, Italy

**Keywords:** acupuncture, auriculotherapy, dysphagia (swallowing disorder), severe acquired brain injuries, tracheal cannula, rehabilitation

## Abstract

Neurogenic dysphagia affects up to 99% of patients with severe acquired brain injury (sABI) upon admission to intensive rehabilitation units (IRU) and increase clinical complications, resource use, and mortality. Conventional dysphagia rehabilitation often relies on active techniques, which are hardly applicable in non-collaborative patients, as is often the case with patients after sABI. In the absence of guidelines for dysphagia rehabilitation that consider the heterogeneity and clinical complexity of these patients, acupuncture and auriculotherapy (AA) could represent a valid intervention to be introduced into clinical practice. The aim of this multicenter double-blind, randomized, controlled trial is to evaluate the superiority of AA combined with conventional rehabilitation compared to conventional rehabilitation alone in improving dysphagia in non-cooperative vascular sABI patients. To this end, improvement in swallowing will be measured using both a clinical scale (Mann Assessment of Swallowing Ability-MASA) and a fiber optic endoscopic assessment (Pooling Score). Certain characteristics of patients admitted to IRUs after sABI, such as the rate and timing of decannulation, the infection rate, and the functional outcome measured by the Glasgow Outcome Scale–Extended, will also be taken into consideration as secondary outcomes. By incorporating AA therapy into a personalized rehabilitation program, this study will examine its potential clinical benefits on dysphagia and the feasibility of this approach in the setting of early intensive rehabilitation for patients with sABI. AA could represent a low-cost, accessible bedside treatment complementary to standard rehabilitation, with no adverse effects, applicable in different clinical settings and at all stages of rehabilitation.

**Clinical trial registration:**

https://clinicaltrials.gov/ct2/show/NCT06888219, NCT06888219.

## Introduction

Neurogenic dysphagia, defined as a swallowing disorder resulting from neurological disease, is a common complication following severe acquired brain injury (sABI) (1, 2). Its prevalence in this population has been estimated at approximately 93% and may reach up to 99% in patients with disorders of consciousness (DoC) (3) at admission to intensive rehabilitation units (IRUs) (4). In patients with sABI, dysphagia increases the risk of malnutrition and dehydration and is associated with higher morbidity, longer hospital stays, and increased healthcare costs (5–8) so enteral tube feeding is commonly employed to mitigate these risks (9, 10). However, although some swallowing disorders are clinically apparent, silent aspiration—defined as aspiration in the absence of a cough reflex—may occur in up to 50% of patients after sABI (11) and can even arise without oral intake, thereby increasing the risk of respiratory infections and mortality (8).

Swallowing disorders are recognized by the World Health Organization as a medical disability with major psychological and social consequences, negatively affecting quality of life (12) and the rehabilitative management of dysphagia represents therefore a central goal during the stay in the IRU. In patients with sABI who remain in a DoC or evolve into a confusional state (13) this goal is often limited by poor cooperation. Conventional rehabilitation approaches (14) require varying levels of patient collaboration, which is frequently compromised in sABI.

It should be noted, however, that patient cooperation is not necessary to achieve oral feeding. Several studies have examined the impact of cognitive level on oral feeding recovery (15). While a cognitive threshold equal to or higher than level 4 on the Rancho Los Amigos Cognitive Functioning Scale (LCF) was initially deemed necessary (2), more recent studies have extended the possibility of oral feeding recovery to patients in a minimally conscious state (16, 17). According to the latest evidence, only patients with unresponsive wakefulness syndrome are very difficult, if not impossible, to rehabilitate with respect to dysphagia (18, 19). Another factor closely related to swallowing that should be taken into consideration during dysphagia rehabilitation in this patient population is the presence of a tracheal tube. Fifty to 70 % of patients with sABI are tracheotomized at the time of transfer to the IRU (4), and its presence has been shown to be closely related to dysphagia in a bidirectional manner (20–22). Tracheotomy can affect the physiology of swallowing, while impaired saliva management and persistent dysphagia remain major obstacles to successful decannulation, even in the long term (23). The inability to swallow saliva can lead to iatrogenic sialorrhea or excessive saliva accumulation (24), resulting in stagnation in the oral cavity that promotes excessive salivation, skin irritation, choking, impaired oxygenation, and even life-threatening pneumonia (25).

Given the clinical complexity and heterogeneity that characterizes this population, the rehabilitation of dysphagia in patients with sABI outcomes represents a considerable challenge (26).

In this context, acupuncture and auricular (AA) therapies emerge as innovative therapeutic options, applicable even in non-collaborative patients, including those in a minimally conscious state and with tracheal tubes, and are grounded in neuroscientific principles aimed at promoting the recovery of swallowing (27). Although a substantial body of evidence supports the potential of acupuncture as a complementary treatment to improve swallowing function, current studies and reviews focus primarily on post-stroke patients (28–32), while evidence in sABI remains limited. Some exploratory studies in patients with traumatic brain injury or DoC have investigated acupuncture-based interventions, reporting indications of possible improvements in arousal or oro-facial responsiveness (33, 34), although findings remain preliminary. In addition, a small number of Chinese clinical studies have examined acupuncture techniques for dysphagia after craniocerebral injury, describing potential benefits in selected patient groups (35, 36). Despite growing interest in AA for dysphagia, no randomized controlled trials have assessed its efficacy in sABI patients with tracheal cannulas and severe dysphagia. This trial aims to evaluate acupuncture plus auriculotherapy in severely dysphagic patients with vascular sABI admitted to an IRU.

## Materials and methods

### Study design, setting and participants

This multicenter, longitudinal, randomized, controlled, patient- and assessor-blinded trial (ClinicalTrials.gov: NCT068882) aims to evaluate whether combining AA with standard logopedic therapy is superior to usual care alone for rehabilitating dysphagia in vascular sABI patients with a tracheal cannula. The protocol was developed in accordance with the SPIRIT 2025 (Standard Protocol Items: Recommendations for Interventional Trials) guidelines (37) and STRICTA (Standards for Reporting Interventions in Clinical Trials of Acupuncture) recommendations (38). Patients admitted to the IRU of the IRCCS Fondazione Don Carlo Gnocchi in Florence will be considered for screening. Eligibility will be determined based on predefined diagnostic, inclusion, and exclusion criteria as outlined below.

#### Diagnostic criteria

- sABI (39) of vascular etiology (ischemic or hemorrhagic), ascertained by Computerized Tomography or Magnetic Resonance examination.
- Severe dysphagia, documented by a Food Oral Intake Scale (FOIS) score = 1.
- DoC or non-collaborative state, defined by LCF ≤ 4.

#### Inclusion criteria

- Time between acute event and enrolment <3 months.
- Age >18 years.
- Presence of tracheal cannula at study enrollment.
- Signature of informed consent by legal representative.

#### Exclusion criteria

- Instability of general clinical conditions (mechanical ventilation, sedation, sepsis, sub-emergent epileptic seizures).
- UWS, according to the Coma Recovery Scale – Revised (CRS-R) (40).
- Known fear of needles (agophobia), as reported by the patient’s legal representative.

**Figure 1:**
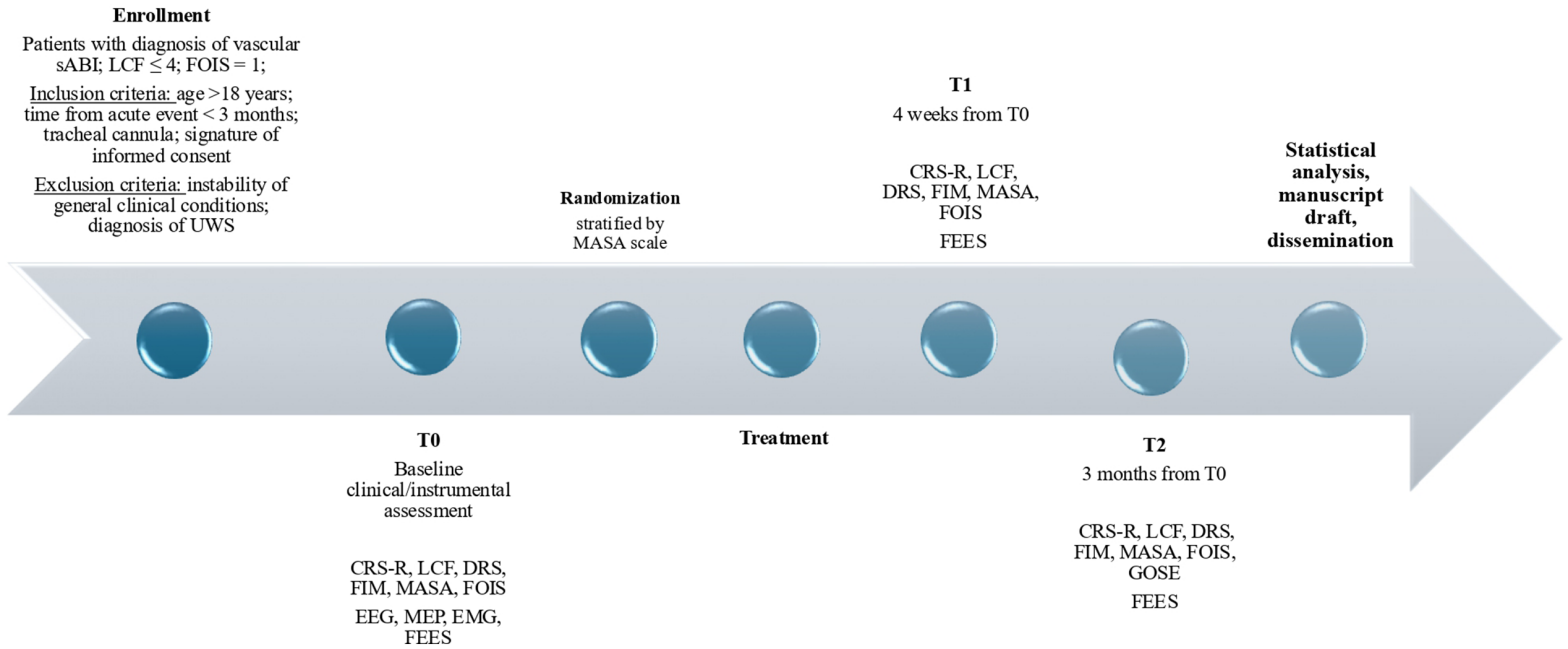
Study timeline. sABI, severe acquired brain injury; LCF, level of cognitive functioning; FOIS, food oral intake scale; UWS, unresponsive wakefulness syndrome; CRS-R, coma recovery scale – revised; DRS, disability rating scale; FIM, functional independence measures; MASA, Mann assessment ofswallowing ability; EEG, electroencephalography; MEP, motor evoked potentials; EMG, electromyography; FEES, fiberoptic endoscopic evaluation of swallowing; GOS-E, Glasgow outcome scale-extended.

Patient’s legal representatives will receive written and oral information about the study aims, interventions, potential risks, and rights. Informed consent will be obtained before enrollment. All subjects will undergo an initial evaluation (T0), an evaluation at 4 weeks (T1) and a follow-up at 3 months (T2) (see Figure 1).

### Clinical and instrumental assessments

Assessments will be performed at recruitment (T0), after the 4 weeks (T1) and after 12 weeks (T2).

Clinical evaluation (planned at T0, T1 and T2) will be supported by the clinical scales recommended by the Italian Society of Physical and Rehabilitation Medicine (41) and includes the CRS-R and the LCF, the Disability Rating Scale (DRS) and the Functional Independence Measures (FIM). Dysphagia will be assessed by the Mann Assessment of Swallowing Ability (MASA) Scale and the FOIS. At T2, the Glasgow Outcome Scale-Extended (GOSE-E) (42) will be performed.

The instrumental evaluations will include the Fiberoptic Endoscopic Evaluation of Swallowing (FEES) (43) integrating the compilation of the Pooling score.

To identify possible confounding factors such as Epileptic status and Intensive Care Unit-Acquired Weakness may interfere with the swallowing function, other data will be recorded from instrumental exams included in the routinary clinical practice (standard electroencephalography-EEG and Electroneuro/myography-ENG/EMG) (44).

Finally, the decannulation achievement and its timing and the infection rate will be recorded.

For the purposes of this study, infections will be recorded as clinical events requiring initiation or escalation of systemic antibiotic therapy supported by clinical signs and, when available, microbiological sampling. Pneumonia will be recorded when respiratory infection is the primary suspected source and is supported by: (i) new or progressive and persistent pulmonary infiltrate/consolidation on chest imaging; (ii) at least two of the following: fever or hypothermia, leukocytosis/leukopenia, purulent respiratory secretions or increased suctioning needs, worsening gas exchange/increased oxygen requirement, or new/worsening cough/dyspnea/tachypnea; with (iii) microbiological support when available (e.g., positive culture from respiratory secretions).

All evaluations will be performed by professionals blinded to the treatment/control arm. Moreover, patients enrolled in the study, given their state of unconsciousness and behavioral/cognitive confusion, may be considered blinded for the purpose of the received AA treatment.

### Randomization, allocation concealment and blinding

**Figure 2:**
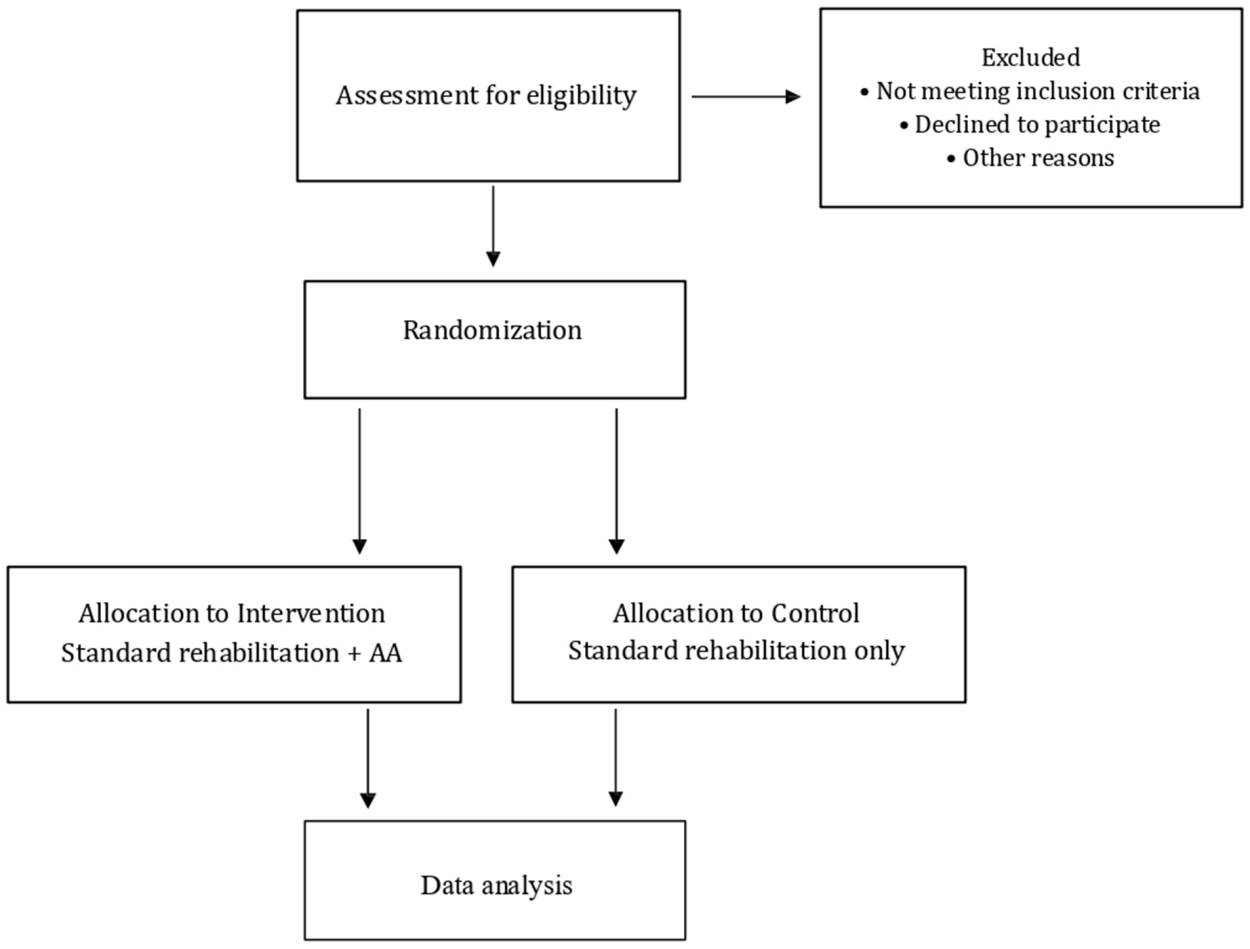
CONSORT-SPIRIT flow diagram. AA, acupuncture + auriculotherapy.

After enrollment, patients will be allocated to one of two groups by computerized randomization and stratified by MASA scale, with an allocation ratio of 1:1, and assigned to either the control or intervention arm. Allocation concealment will be ensured via sequentially numbered, opaque, sealed envelopes prepared by an independent statistician (see Figure 2). The study is designed as double-blind: all clinical and instrumental assessments will be performed by professionals blinded to the treatment arm, and patients are considered blinded either because they present a DoC or due to severe cognitive impairment with short-term memory deficits, which prevent them from being aware of or recalling the treatment received. Due to the nature of the acupuncture intervention, blinding of the acupuncturist is not feasible and represents a potential source of performance bias. To minimize its impact, several mitigation strategies were implemented *a priori*. Acupuncturists will not be involved in outcome assessment, rehabilitation planning, or data analysis. The intervention will be delivered according to a standardized protocol, including fixed session duration, predefined acupuncture points, and uniform treatment setting. Outcome assessors and the rehabilitation team will remain blinded to group allocation throughout the study. Any accidental unblinding will be documented.

Patients assigned to an experimental treatment will undergo 10 sessions of AA in addition to conventional treatment for a total duration of 4 weeks. Those assigned to the control group will undergo only conventional treatment.

### Conventional rehabilitation

All professionals involved in the rehabilitation of enrolled patients will be blinded to the study’s arm. Regardless of the arm of the study, all patients will undergo a customization based on the rehabilitation team rehabilitation plan and will include speech and physiotherapy.

**Figure 3:**
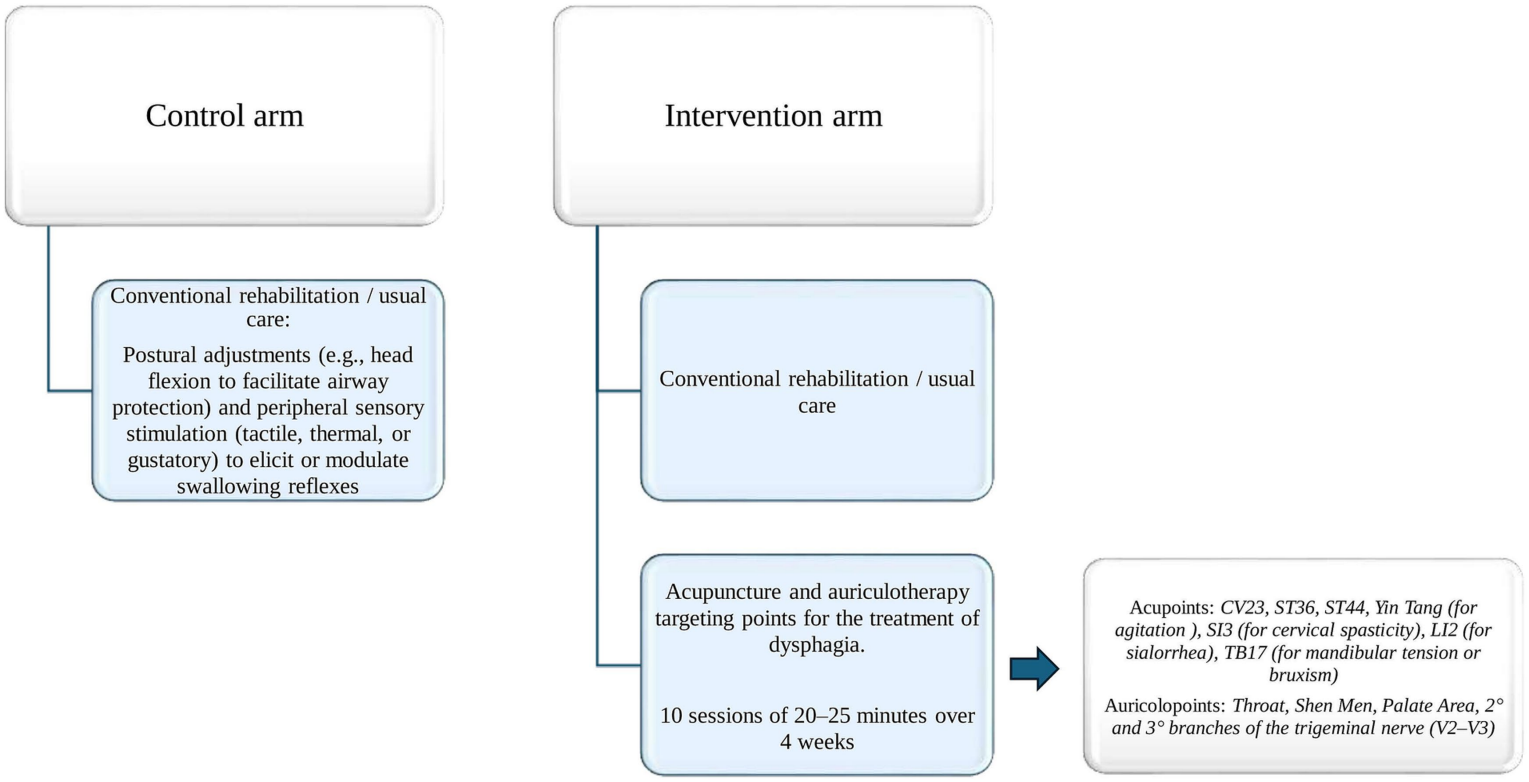
Intervention protocol. CV23, Conception Vessel 23; ST36, Stomach 36; ST44, Stomach 44; SI3, Small Intestine 3; LI2, Large Intestine 2; TB17, Triple Burner 17; V2–V3, 2° and 3° branches of trigeminal nerve.

Dysphagia rehabilitation will consist in passive approaches, including postural adjustments (e.g., head flexion to facilitate airway protection), oropharyngeal and peri-oral sensory stimulation—tactile, thermal, or gustatory—to elicit or modulate swallowing reflexes (45, 46), respiratory and orofacial facilitation, and oral hygiene protocols to prevent aspiration-related complications. Whether applicable, active techniques, such as the Shaker maneuver (47), Chin Tuck Against Resistance (48) or specific swallowing maneuvers like the supraglottic swallow, effortful swallow and the Mendelsohn maneuver, will be used based on the level of collaboration reached during the three-month period of experimentation (see Figure 3).

### Intervention

Patients assigned to the intervention arm will receive a total of 10 combined AA sessions lasting 25 minute each, in addition to the conventional treatment mentioned above. In order to balance practical feasibility, patient tolerance and clinical relevance in the context of early in-patient intensive rehabilitation, the AA treatment protocol was designed taking into account both the operating model applied at the Regional Reference Center for TCM “Fior di Prugna” and the length of stay in intensive rehabilitation wards, where the average hospital stay is approximately 4 weeks. Each session will be performed with the patient supine in bed or seated in a wheelchair by the acupuncturist qualified by a school registered to the Italian Federation of Acupuncture Societies. Sterile disposable acupuncture needles will be used.

The acupuncture protocol includes somatic points based on their traditional indications in Chinese traditional medicine and their relevance to the neuromotor and autonomic dysfunctions underlying swallowing disorders. Zusanli (ST36) and Neiting (ST44), both on the Stomach meridian, for their roles in digestive regulation and inflammation control. ST36 is known for enhancing gastrointestinal function, modulating systemic inflammation, and supporting motor recovery, while ST44 may help reduce oral inflammation and sialorrhea (49). Lianquan (CV23), traditionally indicated for tongue and throat disorders and widely used to facilitate articulation and swallowing by targeting the suprahyoid and lingual muscles (50). Additional points will be selected based on individual needs: Houxi (SI3) for cervical spasticity; Yifeng (TB17) for mandibular tension or bruxism; Yintang (EX-HN3) for agitation and to promote treatment cooperation, and Erjian (LI2) for sialorrhea, due to its role in clearing internal heat and regulating fluids.

The auricular acupuncture protocol was developed based on neuroanatomical rationale and preliminary clinical evidence supporting the role of cranial nerve stimulation in swallowing rehabilitation. The selected points—Throat, Shen Men, Palate Area, and zones corresponding to the second and third branches of the trigeminal nerve (V2–V3)—were chosen to engage key sensory and motor pathways involved in swallowing control (50). Stimulation of the throat point—anatomically corresponding to the pharyngeal region and innervated by trigeminal branches—has been associated with facilitation of the pharyngeal reflex; randomized trials using magnetic pressure have demonstrated promising outcomes in poststroke dysphagia (51). The Palate Area is involved in the preparatory and oral phases of swallowing. Its proximity to zones innervated by the facial and trigeminal nerves supports its role in enhancing oral motor coordination and bolus transit. The V2–V3 auricular zones were included for their involvement in orofacial motor control, mastication, and sensory feedback from the oral cavity. Stimulating these areas may improve oral awareness and functional control during the early stages of swallowing. Shen Men, while not directly connected to oropharyngeal structures, was included for its autonomic and limbic modulatory effects. It has been shown to reduce stress and sympathetic overactivation, which can impair swallowing coordination, particularly in patients with cognitive or emotional dysregulation post-injury (52) (see Figure 3).

### Harms and adverse events

Expected adverse events (AEs) include mild pain, local bleeding or bruising at needle sites. Serious adverse events (SAEs) can include pneumothorax. All AEs and SAEs will be documented from randomization to the end of follow-up, with causality assessment by the investigator. No thoracic points will be used to prevent pneumothorax. To prevent the inadvertent retention of needles, the acupuncture physician will perform a systematic needle count at the end of each procedure.

### Adherence and co-interventions

Session attendance will be recorded. Participants missing >30% of sessions without medical justification will be considered non-adherent. Other neuromodulatory therapies (e.g., pharyngeal electrical stimulation, rTMS) will not be permitted during the trial.

### Discontinuation and modification criteria

Intervention discontinuation will occur in cases of clinical deterioration, adverse events judged to be treatment-related or withdrawal of consent by the legal representative.

### Outcome measures

The primary outcome is the improvement of dysphagia and will be measured using the MASA scale (53, 54) and the instrumental scale “Rationale scores based on endoscopic landmarks and bedside parameters” with relative values (43) at T1. The same scales will be used at T2 to measure the maintenance of the effect.

Secondary outcomes include: (1) improvement on the decannulation rate and/or reduction in decannulation timings at T1 and T2; (2) reduction of the rate of infections at T1 and T2; (3) improvement on functional autonomy measured by the GOS-E at T2.

### Statistical analysis

At present, we have designed a pilot study with the purpose of providing preliminary data necessary to calculate the appropriate sample size for a subsequent randomized controlled trial (RCT). The main study will be a 2-arm, randomized, controlled trial for which a medium effect size was conservatively hypothesized. According to the stepped rules of thumb proposed to estimate the size of the pilot based on Cohen’s d effect size (55), for an 80% power main trial, the pilot sample size should be 10 per arm, and the subsequent main trial will require somewhere between 34 and 176 participants per arm. The sample size of 10 participants per arm will be increased to 12 participants per arm to account for potential dropouts. This sample size was determined exclusively since methodological recommendations available in the literature. The data obtained from this pilot study will be used to estimate the effect size and variability, which will inform the power analysis and the determination of the required sample size for the definitive RCT. Analyses will be conducted using SPSS version 28 software (SPSS, SPSS Inc., Chicago, United States). The normality of the distribution of continuous variables will be checked using the Shapiro–Wilk test. If the variables violate the assumption of normality, they will be presented through their median value and interquartile range, otherwise through mean and standard deviation. Categorical variables will be represented through absolute frequencies and percentages. Baseline characteristics will be compared between treatment and control groups using independent *t*-tests or Mann–Whitney U tests for continuous variables, depending on data distribution, and chi-square or Fisher’s exact tests for categorical variables. The primary outcome (improvement in dysphagia) will be assessed by comparing MASA scores and “Pooling score” between groups at T1 using ANCOVA models (adjusting for baseline values) or non-parametric alternatives, if assumptions are not met. Maintenance of the effect at T2 will be evaluated using linear mixed models with random intercepts, including group, time (baseline, T1, T2), and their interaction as fixed effects; if model assumptions are violated, change scores between T1 and T2 will be calculated and compared between groups using independent *t*-tests or Mann–Whitney U tests. Secondary outcomes will be analyzed as follows: decannulation rates at T1 and T2 will be compared between groups using chi-square or Fisher’s exact tests, while decannulation times will be compared using independent *t*-tests or Mann–Whitney U tests and Kaplan–Meier survival analysis. Infection rates at T1 and T2 will be compared between groups using chi-square or Fisher’s exact tests. Functional autonomy at T2, measured by the GOS-E, will be compared between groups using independent *t*-tests if normality assumptions are met, or Mann–Whitney U tests otherwise. A *p*-value <0.05 will be considered significant in all analyses. The primary analysis will follow the intention-to-treat (ITT) principle. Sensitivity analysis will be performed to assess the robustness of the findings, including per-protocol analysis restricted to patients who completed the assigned intervention.

### Data collection and management

Data will be collected through electronic case report forms (eCRFs) using the REDCap platform. Data entry will include built-in logic and range checks to ensure accuracy. All identifying information will be pseudonymized. Access to the database will be password-protected and restricted to authorized personnel only. Data will be stored on secure servers for 7 years after trial completion before anonymization.

### Data monitoring and quality assurance

Monitoring will be conducted by an independent study monitor from the research coordination office of the IRCCS. Source data verification will be performed on at least 10% of cases. Any protocol deviation will be documented and assessed for impact on study integrity.

## Discussion

The rehabilitation of dysphagia in patients surviving a sABI presents a significant challenge, particularly when initiated in the early phase in which patient cooperation is minimal or absent. In such cases, speech-language rehabilitation techniques requiring active patient participation are not feasible. Instead, passive or neuromodulation-based approaches must be considered. Within this context, acupuncture and auriculotherapy emerge as promising treatment options since these therapies do not rely on patient cooperation. The main purpose of this study is to determine whether acupuncture and auriculotherapy can serve as an appropriate and effective treatment for rehabilitating dysphagia in non-communicative patients surviving a sABI.

The present study protocol has been carefully designed to consider not only the individual characteristics of dysphagia, the patient population, and the proposed intervention, but also the dynamic interactions among these three factors. An accurate assessment of swallowing function represents a critical step for monitoring the primary outcome namely the progression of dysphagia throughout the rehabilitation process. For this purpose, in addition to FOIS offering a crude description of dysphagia, the MASA, a bedside clinical scale for swallowing consisting of 26 items that can be easily administered by speech therapists, was chosen. This scale has demonstrated good psychometric properties in brain-injured patients with cognitive impairment (53) and has been used in tracheostomized patients (56). As is often the case with clinical scales, the reliability of the MASA can be reduced in non-communicative patients because some items—such as auditory comprehension, expressive communication, alertness, cooperation, command following, apraxia, articulation, oral preparation and transit, and respiratory-swallow coordination—can be challenging to administer. To mitigate this limitation, an instrumental evaluation by FEES to detect any changes—including subclinical ones—in swallowing function is included in this study. This dynamic endoscopic technique primarily evaluates the pharyngeal phase of swallowing—excluding the whiteout period—while also providing indirect information about the oral and esophageal phases. Furthermore, FEES allows for the targeted assessment of laryngeal sphincter function, sensory responsiveness, and the presence of pharyngeal residue. Its minimally invasive nature and feasibility for bedside application make it particularly suitable for patients in unstable clinical conditions (57). Given the complexity of these cases, which often involve reduced responsiveness and impaired postural control, FEES is strongly recommended both to guide the initiation of oral feeding rehabilitation programs (58, 59) and to support decisions regarding decannulation The Pooling Score, a clinical endoscopic evaluation will be used to assess the severity of swallowing disorders, considering excess residue in the pharynx and larynx (43). This score ranges from a minimum of 4 to a maximum of 11 and is calculated by summing the scores assigned to: Bolus location, Amount of residue, Ability to control residue/bolus pooling, assessed based on coughing, throat clearing (raclage), and the number of dry voluntary or reflex swallowing attempts (<2, 2–5, >5).

In addition to the characteristics of dysphagia itself, several peculiarities of sABI patients—such as functional status, neck posture in a seated position, muscle tone, level of consciousness, and cognitive function—must be carefully considered, as they can significantly influence its severity. For this reason, a precise multidisciplinary assessment was incorporated into the selection of evaluation scales (DRS, FIM, CRS-R, LCF). The CRS-r (60, 61) is the main current clinical assessment tool for the behavioral diagnosis of DOC, while the LCF is widely used in monitoring the progression of cognitive recovery, with lower scores (LCF 1–3) for levels of impaired consciousness, and progressively higher scores for levels of increased cognition. To support and complement the clinical evaluation, some instrumental examinations routinely practiced in the in-patient ward were included in the study. EEG, EMG, and MEP will be performed to objectively assess potential neurological deficits or potential confounders factors, such as epileptic status, severe hyposthenia involving trunk muscles, severe critical illness neuromyopathy. Additionally, the inclusion criteria were selected based on scientific evidence and the expected clinical impact. For instance, patients in an UWS were excluded considering recent findings indicating the absence of the oral phase of swallowing in this population (16). Also, particular attention was given to minimizing heterogeneity within the study population by including only patients with a vascular etiology.

For what concerns the intervention modalities, the protocol, including the duration and frequency of treatment, was developed following a thorough review of the literature on the use of somatic and auricular acupuncture in the rehabilitation of dysphagia in patients with central neurological damage. Acupuncture, a key component of TCM, involves the insertion of fine needles with manual or electrical stimulation (62) to restore qi balance along meridians (63). Literature suggests its effectiveness in managing neurological symptoms such as spasticity and pain (64) and some randomized controlled trials reported benefits for post-stroke dysphagia (65, 66). Although, a Cochrane review found insufficient evidence due to high study heterogeneity (32), acupuncture has been recently recommended with moderate evidence by the European Stroke Organization and the European Society for Swallowing Disorders for post-stroke dysphagia treatment (30).

Auriculotherapy involves stimulation of specific points on the auricular pavilion, considered a major microsystem of the body (67). From a Western medical perspective, its effects are understood through neurophysiological and reflexogenic mechanisms (67), as the ear receives extensive innervation from four mixed cranial nerves—trigeminal, facial, glossopharyngeal, and vagus nerve—as well as from the cervical plexus (C2/C3) (68). To date, no studies have specifically investigated auriculotherapy in patients with sABI, and only limited evidence exists regarding its use in post-stroke dysphagia. Together, these points provide a multi-target approach that combines peripheral sensorimotor facilitation with central autonomic modulation. This strategy aligns with evidence suggesting that auricular stimulation may activate brainstem nuclei—such as the nucleus tractus solitarius and nucleus ambiguus—and support neuroplasticity in neurorehabilitation. The inclusion of multiple auricular points in our protocol was also motivated by practical considerations related to the clinical condition of sABI patients. Severe motor impairments, abnormal postures, spasticity, and involuntary movements often hinder access to body acupuncture points, compromising safety and feasibility. In contrast, the auricle provides a compact, easily accessible surface where stimulation can be safely administered with minimal repositioning, even in bedridden or low-responsive patients. This anatomical and logistical advantage makes auriculotherapy particularly well-suited for complex neurorehabilitation contexts. Finally, the effectiveness of somatic and auricular acupuncture will be evaluated using a treatment schedule aligned with evidence-based protocols reported in the literature (29). The persistence of treatment benefits will be assessed at follow-up (T2), conducted 2 months after the conclusion of acupuncture therapy, during which patients will continue to receive standard rehabilitation care.

## Conclusion

Dysphagia is a significant disability for patients with sABI, and its management is crucial, particularly in the early stages of rehabilitation. Acupuncture and auriculotherapy offer promising avenues to enhance interdisciplinary care for these patients, being cost-effective, easily administered at the bedside, associated with minimal side effects and without adding pharmacological burden. This randomized double-blind trial proposes to explore the clinical effectiveness and feasibility of these techniques as part of an integrated rehabilitation approach.
